# Mechanically Tunable Flexible Photonic Device for Strain Sensing Applications

**DOI:** 10.3390/polym15081814

**Published:** 2023-04-07

**Authors:** Murad Ali, Muhammad Waqas Khalid, Haider Butt

**Affiliations:** 1 Department of Mechanical Engineering, Khalifa University of Science and Technology, Abu Dhabi 127788, United Arab Emirates; haider.butt@ku.ac.ae; 2 School of Engineering, University of Birmingham, Birmingham B15 2SQ, UK

**Keywords:** photonics devices, PDMS, surface imprinting, diffraction pattern, optical diffusion

## Abstract

Flexible photonic devices based on soft polymers enable real-time sensing of environmental conditions in various industrial applications. A myriad of fabrication techniques have been established for producing optical devices, including photo and electron-beam lithography, nano/femtosecond laser writing, and surface imprinting or embossing. However, among these techniques, surface imprinting/embossing is simple, scalable, convenient to implement, can produce nanoscale resolutions, and is cost-effective. Herein, we utilize the surface imprinting method to replicate rigid micro/nanostructures onto a commonly available PDMS substrate, enabling the transfer of rigid nanostructures into flexible forms for sensing at a nanometric scale. The sensing nanopatterned sheets were mechanically extended, and the extension was remotely monitored via optical methods. Monochromatic light (450, 532, and 650 nm) was transmitted through the imprinted sensor under various force/stress levels. The optical response was recorded on an image screen and correlated with the strain created by the applied stress levels. The optical response was obtained in diffraction pattern form from the flexible grating-based sensor and in an optical-diffusion field form from the diffuser-based sensor. The calculated Young’s modulus in response to the applied stress, measured through the novel optical method, was found in a reasonable range compared to the reported range of PDMS (360–870 kPa) in the literature.

## 1. Introduction

Soft and flexible optical sensors are emerging as the new class of transducers, being considered as an alternative to conventional electronic devices [1]. Electronic sensors are composed of active components which require a power supply to function and have limitations such as a high cost and manufacturing complexity. The efficiency of such sensors is also affected by electromagnetic (EM) and thermal noise interference [2], making them unusable in hazardous environments. Polymerized nano/microstructures-based optical components are used to detect and monitor environmental changes such as humidity, pressure, shear, and torsion—having potential applications in robotics, wearable devices, medical diagnostics, and healthcare monitoring [3,4,5,6,7,8]. Flexible optical sensors have been utilized in temperature and strain quantification due to their low cost, compactness, being immune to thermal or EM noise, high sensitivity, and reliability [9,10,11]. Therefore, developing simple, cost-effective, and robust optical sensors is highly desirable for remote-sensing applications.

Many fabrication techniques produce micro/nanostructural elements for sensing applications, including micro-electromechanical methods, nano-imprinting, photo and electron-beam lithography, FIB milling, and direct laser writing [12,13,14,15,16,17]. However, there is an increasing interest in finding alternative approaches to these sophisticated fabrication techniques due to their high operation/maintenance costs, expertise-dependence, and material limitations. Recently, fast and low-cost holography techniques have been used to fabricate miniature optical devices (lenses, diffusers, and gratings) [18,19,20]. The soft lithography-based surface imprinting fabrication/replication method is relatively lower cost, with an uncomplicated setup, high throughput, and more appealing pattern resolution in the micro to the nanoscale with high precision [21,22].

Periodic gratings have been extensively used in a wide range of applications such as data storage [23], light trapping [24], security [25], holographic displays [26,27], and biosensing [28,29]. Under specific light illumination, periodic gratings diffract light, depending on their periodicity and the wavelength of the incident light. Diffraction angles or the angular separation between the diffracted orders and the central non-diffracted spot can be altered by applying an external stimulus (detected using Bragg’s law) [15,30]. Various detection schemes that include diffraction efficiency [26,31], intensity [32,33], and wavelength shift [34] can be used to obtain useful data for stimulus detection. The involvement of dimensional physical change in these optical sensors emphasizes the usage of flexible and stretchable materials for the fabrication of the sensing gratings [31,34,35,36].

The monitoring of the strain or force field is important in many industries such as aerospace, nuclear power stations, and mining to monitor any undesirable stresses leading to damage to the radioactive storage containers and other equipment or within the region of interest. Conventionally, this is realized by many conventional wireless or cabled devices such as extensometers and strain gauges which are expensive, incompatible with harsh environments, require ruggedized casing, and are also made with active components requiring a power supply to function [37,38]. Therefore, the need to fabricate flexible and passive photonic devices for strain measurement and monitoring is ubiquitous. For instance, other applications may include human motion detection, human joint motion monitoring, and implantable devices [39,40].

Here, we replicated two different optical structures, namely diffraction gratings and engineered diffusers via the nanoimprinting method on polydimethylsiloxane (PDMS) sheets, in order to examine the strain and force fields. PDMS is the most extensively used elastomeric material used in micro throttle pumps [41], catheter surfaces [42], wound dressings and bandages [43], piezoelectric microvalves [44], and optical systems [45]. It is classified as silicones, belonging to a group of polymeric organosilicon compounds [46]. PDMS can be used between −50 °C to 200 °C [47,48], has a low elasticity (360–860 kPa) [49,50], and low absorption losses within the range from kPa to MPa [51]. Low cost [52], high stretchability [53], low-surface energy [51], and biocompatibility [53] make this easily available soft elastomeric material suitable for prototype testing [52]. The material is compatible with traditional micro-processing and soft-stamping processes, making it attractive to be used as a primary or secondary mold [47,54]. Due to its elastic properties, PDMS can withstand up to 140% reversible stretch depending upon its thickness [55]. This organosilicon material offers a good transparency (up to 90%) in a wide spectral range (350–950 nm) which proves its feasibility in examining the mechanical strain by optical methods [56,57].

### The Diffraction-Based Optical Sensing Scheme

The transmitted laser beam from the periodic gratings will be diffracted based on the periodicity of the gratings, yielding a uniform diffraction pattern on the image screen. The diffraction pattern is composed of a more intense non-diffracted central spot along with diffracted orders. Based on the distance between the central spot to the first or higher order, the relevant diffraction angle can be calculated using Equation (1) [58,59,60,61].

(1) $$\sin\theta =\frac{l_{1}}{\sqrt{l_{1}{}^{2}+l_{2}{}^{2}}}$$

where θ represents the diffraction angle, $l_{1}$ represents the first-order interspace, and $l_{2}$ represents the distance between the grating sample and the image screen. The following equation can calculate the relationship between the grating period/periodicity and the separation between the diffracted orders and the non-diffracted central spot.

(2) $$d_{1}=\frac{k\times \lambda }{\sin\theta }$$

In order to calculate the distance of the first diffraction, the value of k can be set as 1, while  λ is the wavelength of the incident light. Furthermore, the strain created by any specific external stimuli (force/stress, temperature) depends on the characteristics of the material in which the optical structure is engraved. The length of the grating sample increases with increasing applied stress, which in turn lengthens the periodicity of the grooved gratings. Using the extension ratio, the grating period from a macro-perspective can be calculated as follows:

(3) $$d_{2}=d_{0}\times \left(1+\eta \right)$$

where $d_{0}$ represents the original grating period, and η is the extension ratio or total applied strain which can be calculated by measuring the first-order interspace. The change in optical response can then quantify the strain produced by specific values of the applied stress. However, optical diffusers comprise micro/nanostructures, which enable incident sharp laser beams to scatter at wide angles producing uniform diffusion patterns. The optical response, i.e., diffused light at specific angles, depends upon the wavelength of the transmitted light and the surface structures of the diffuser, underhand in a similar manner to the diffraction gratings. Diffusion angles and light transmission can be varied by manipulating the diffusing surface structures with the applied stress.

## 2. Materials and Methods

### 2.1. Materials and Equipment

A rectangular 1D grating pattern was purchased from the JunAN (SL150-18, China) and used as a mold during the replication process. A silicone elastomer base and curing agent (SYLGARD 184, 1.1 kg), purchased from Farnell, Leeds, UK, were used as a target material to achieve micro/nanostructures in a flexible form. An optical spectrophotometer (resolution of −0.1–100 nm FWHM) was purchased from Ocean Optics for the optical measurements. Optical spectrophotometer, fabricated flexible periodic gratings, flexible optical diffusers, and broadband/monochromatic light sources were used to conduct the experiments for the optical characterization of our samples. The monochromatic light sources: blue (450 nm, 2.6 mW, Ø11 mm), green (532 nm, 4.5 mW, Ø11 mm), and red (405 nm, 4.5 mW, Ø11 mm) lasers were purchased from Thorlabs Elliptec GmbH (Dortmund, Germany). The finite element tool COMSOL Multiphysics 5.2 and MATLAB (Math Works, R2013, Natick, MA, USA) were used for the numerical simulations and data processing.

### 2.2. Fabrication of Flexible Nano/Microstructures

**Figure 1:**
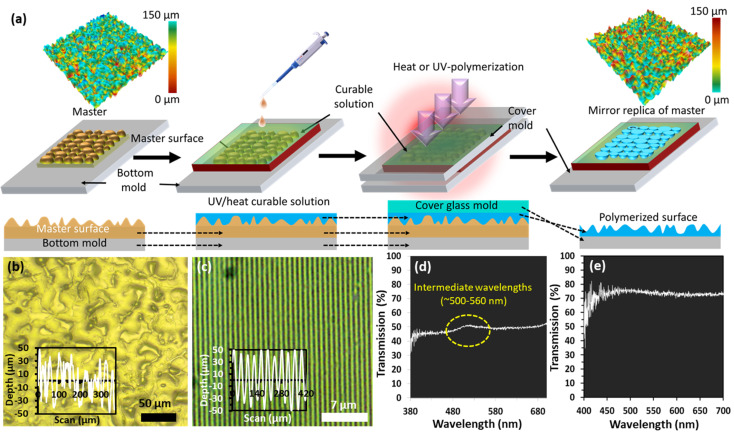
Micro/nano fabrication process (mirror replication) of selected master patterns on PDMS: (**a**) schematic representation of transferring rigid structures (micro/nanostructures) onto flexible substrates; (**b**,**c**) optical images representing morphological surface analysis of the polymerized flexible optical diffuser and diffractive grating surfaces, respectively; optical transmission spectra of replicated (**d**) flexible optical diffuser and (**e**) diffractive grating surface in the wavelength range of (380–700 nm) using a broadband (white light) source.

Flexible polymerized optical elements (based on nano and microstructures) were fabricated using a soft stamping/molding process (Figure 1a). PDMS is a type of polymer that is based on silicon. It consists of an elastomer base and a curing agent, acquired from Farnell, UK. The most common mixing ratio for PDMS is 10 parts Sylgard-184 silicone elastomer base to 1 part Sylgard-184 silicone elastomer curing agent [62]. The PDMS polymer solution was prepared by mixing the PDMS monomer and the curing agents (10:1, volume per volume (*v*/*v*)). The mixture was mixed and stirred for 20 min, followed by ultrasonication to remove trapped air bubbles from the mixture. The master stencil of periodic gratings or diffusing surface was placed in a Petri dish. The PDMS liquidized mixture was poured directly on the stencil fixed in the dish and spin-coated at 400, 600, and 800 rpm for uniform distribution on the top of the stencil mold. Samples were cured at 50 °C for three hours on a hotplate and then detached from the master stencil.

### 2.3. Optical Characterization and Simulation

The fabricated diffuser and grating surfaces were characterized for surface morphology and optical transmission using an optical microscope (Zeiss, 20, 50×, Jena, Germany). Samples fabricated in this manner were directly used for sensing applications through a customized optical characterization setup. The samples were fixed on a computer-controlled 2D rotational stage and illuminated typically by monochromatic light, blue (450 nm), green (532 nm), and red (635 nm) light, as well as by broadband light (from Thorlabs, Newton, NJ, USA). The far-field optical response was recorded on an image screen placed at a distance of 12 cm in front of the sample to determine the interspacing between the first order and the zero order for the periodic grating sample, while the diffused light pattern was imaged for the diffusing surface sample. The image screen was replaced with a photometer to record the angle-dependent intensity measurements. To experiment with our samples for various stress levels, one side of the sample (film with rectangular 1D grating pattern) was kept fixed while the second side of the sample was attached to a holder to gradually load different known weights to obtain the resulting stress (kPa) and strain, respectively. The optical response for each strain value was recorded from the image screen and correlated to the related Young’s modulus.

The finite element method (FEM) is a rapid approach for modeling various designs and materials through software. It can solve problems with a higher degree of precision, is useful for simulations that involve time or frequency dependencies, and can utilize boundary conditions (such as point forces, distributed forces, and thermal effects) to define the model’s response. As a result, FEM was employed to carry out computational modeling of the 1D diffraction gratings. The COMSOL Multiphysics software, which is commercially available and based on FEM, was utilized for optical modeling. This software is easy to use, with a user-friendly graphical interface that allows for the modeling of small-scale diffractive optical devices and structures. It includes features such as 2D/3D modeling, user-defined variables, material selection, customized or variable grid/mesh sizes, and accurate results analysis in the time or frequency domain with smaller time periods.

The flexible diffraction grating was modeled using the COMSOL Multiphysics software. The optical diffraction properties of the grating were simulated using broadband light that was directed at the polymer material-based rectangular grating surface at a normal incident angle. The effect of strain on the grating period was simulated by expanding the rectangular structure by 10%, 20%, and 30%. The transmitted light from the rectangular grating structure was measured in an air medium. A user-defined, physics-controlled mesh with a mesh size of one-fourth of the wavelength was used for modeling. The mesh was customized to contain more elements around the spherical domain, and a swept mesh was applied to reduce the number of elements and complexity of the simulation. Customizing the meshing helped to reduce the memory requirements by creating an efficient and accurate simulation environment. The continuity and scattering boundary conditions were considered during the FEM simulation, and sub-meshing and mesh convergence tests were performed to ensure result accuracy. Triangular meshing elements were used in the simulation domain, with a maximum degree of freedom of 135,860. The completed mesh consisted of 705 boundary elements and 18,513 domain elements. The simulation time was 30 s for the 2D simulation and 25 min for the 3D simulation. Therefore, 2D simulation was performed to reduce the computational time and complexity. The flexible diffraction grating was considered as a rectangular grating structure, and any compression or expansion of the structure was treated as a variation in the period of rectangular grating structures.

## 3. Results and Discussion

An optical microscope was utilized to analyze the surface profile of the resulting grating and diffuser samples to confirm the successful pattern replication (transfer of the master surfaces onto the PDMS film). The dimensions of replicated structures were also in good agreement with master nanopatterns. The width (W) of the samples was measured to be around 852.1 µm from the microscopic image, where the period is Λ (700 nm), ridge width is A (350 nm), and D = (A/Λ) is the duty cycle (0.5 or 50%) of the grating structure. The surface roughness of the master pattern before replication was in the range of 0–150 µm, where most of the features were below 150 µm. After fabrication, the surface roughness was slightly higher, and features were mainly close to 150 µm (Figure 1a). The morphology investigation revealed that the polymerized diffuse surface has micron-sized features with an irregular wavy-type morphology responsible for the random scattering of the incident light. The line surface profile of a specific region shows peaks and valleys, evident by features in the range of −40 to 50 µm (Figure 1b). On the other hand, the grating surface exhibited a smoother line profile due to its successful replication, where the peak heights and valley depths of rectangular grating features are on the nanometer scale. In addition, the line profile shows smoother characteristics (−50 to 50 µm from base to height) for the grating sample (Figure 1c). The replicated surfaces were also investigated by optical transmission spectroscopy in the wavelength range of 380–700 nm (Figure 1d,e). As expected, the diffusing surface resulted in more light scattering and allowed around 50% of the incident light to pass through the sample (Figure 1d). On the other hand, due to the high smoothness and uniformity of the periodic grating structure, more incident light was transmitted that led to around 72% average light transmission (Figure 1e).

The optical characterizations of replicated diffusion surfaces were carried out using a customized optical setup to investigate diffusion angles in response to monochromatic and broadband light sources. The setup consisted of an optical power meter and different monochromatic (450 nm (blue), 532 nm (green), and 650 nm (red)) and broadband (white light) light sources. The replicated surfaces were analyzed using a two-dimensional (2D) rotational stage (CR1/M, Thorlabs) powered by a motor and mounted on the optical bench. This stage had a high level of accuracy, with precise 0.5° steps in rotation. It could rotate from 0° on the left side to −90° and from 0° on the right side to +90°. The rotating stage was connected to the sample stage through a connecting rod. On the sample stage, the power meter and a two-axis translational mount were mounted where the samples were able to move in both the x and y directions. The specimen was positioned on a two-dimensional stage, which allowed for free movement along the x and y axes. Monochromatic light sources emitting red (635 nm), green (532 nm), and violet (435 nm) wavelengths, along with broadband light sources, were directed at the specimen perpendicular to its plane. A photometer was fixed at 0° while the two-dimensional stage was rotated from 0° to 180°.

**Figure 2:**
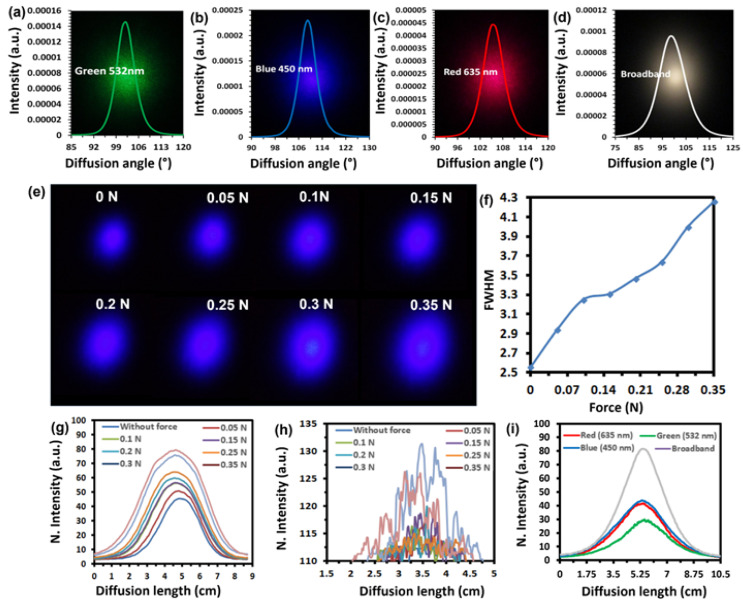
Optical characterization of replicated polymerized diffuser samples. (**a**–**d**) Light intensity distribution profiles of samples in response to three monochromatic lasers (450 nm, 532 nm, and 650 nm) lights and broadband (white light) sources. (**e**) The optical images of the diffusion field were captured from the image screen by illuminating the diffuser sample with a blue laser (450 nm) beam in the applied load range of 0 to 0.35 N. (**f**) FWHM measurements in response to an applied load in the range of 0 to 0.35 N. (**g**) The variations in optical diffusion length of the diffuser sample in response to an applied load in the range of 0-0.35 N using a blue laser light source. (**h**) Measurable fluctuations in intensities around the center at 0 in response to an applied load (0–0.35 N) (**i**) Line intensity distribution profiles across the diffusion area at the image screen using white light and monochromatic lasers (blue, green, and red) sources.

The replicated diffusion pattern was illuminated with three laser light sources (450 nm, 532 nm, and 650 nm), as well as the white light source. The transmitted light spectrum through the diffusing optical structure in light-intensity-distribution form was recorded in the 0°−180° range. In Figure 2a–c, the diffusion angles for the red (650 nm), green (532 nm), and blue (450 nm) wavelengths were recorded as 121°, 114°, and 121°, respectively. The average diffusion angle (without any stress applied) in response to a white light source was recorded to be around 120°, as depicted in Figure 2d.

**Figure 3:**
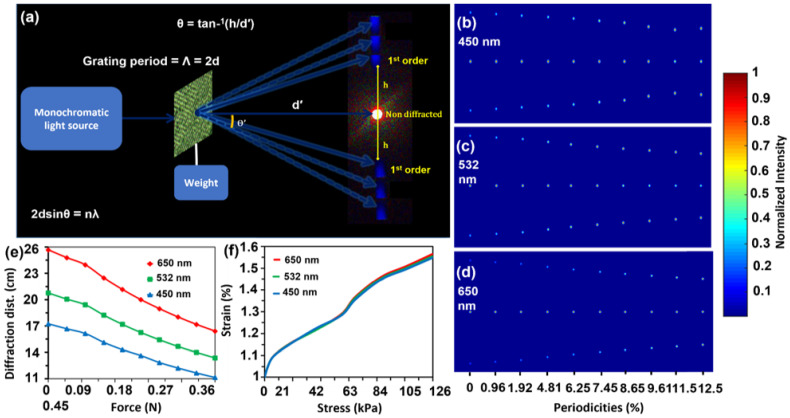
Experimental setup for optical characterization and simulations. (**a**) Schematic of the apparatus setup to perform optical characterization of the polymerized samples; (**b**–**d**) diffraction angles (degree) of the diffracted spots through different periodicities of the periodic gratings (i.e., left to right are for finer to gradually increasing grating spacing) under blue (450 nm), green (532 nm) and red (650 nm) normal illuminations, respectively. (**e**) Plots of diffraction angles against various stress levels under various incident lights (**f**) stress versus strain curves for red (650 nm), green (532 nm), and blue (450 nm) light illuminations.

The average transmitted light (%) through the flexible optical diffuser was recorded to be 50% in the visible range. The diffusion angle measured for the green (532 nm) wavelength was slightly lower than that for red (635 nm) and blue (450 nm) wavelengths and might be attributed to a small measurable peak that appeared in the transmission curve in the −500–560 nm range Figure 1d. Thus, a narrower diffusion field is obtained due to less scattering of transmitted light, i.e., this light interacted less with the structure present in the sample and focused at the center at near 0°. In the next step, the photometer was replaced with the image screen to investigate the change in the optical properties of our sample under various load levels. Experimental arrangements were made similar to the experiment performed for periodic gratings, as shown in Figure 3a. The surface features engraved on the PDMS-based diffuser substrate were distributed randomly, causing light diffusion across wide angles. According to Bragg’s law, diffusion angles are defined by the size of the structures when the dimensions of the structures are comparable to the wavelength of transmitted light. Due to the random distribution of surface structures with various sizes ranging from the nanometer to micrometer scale, the diffusion effects were also based on Rayleigh scattering, where scattered light was more significant than the physical structures in the target [60]. Digital images of the diffusion field were captured using a blue laser (450 nm) in the load range of 0–3.5 N, indicating that the field of view size increased with the increase in applied load and could be attributed to the large scattering of incident light (Figure 2e). Therefore, the measured diffusion length at full width half maximum (FWHM) also increased and varied from 2.56 cm to 4.26 cm for no force to the maximum applied force of 0.35 N, respectively, as shown in Figure 2f. The FWHM is utilized to measure the degree of light diffusion, and the increase in diffusion length with the increase in applied force suggests that a higher degree of diffusion is occurring in the targeted sample. 

The intensity profile of the fabricated optical diffuser with respect to an applied load in the range of 0–0.35 N was obtained (Figure 2g). The normalized peak intensity of the scattered light was recorded as 45.7 (a.u.) to 79.4 (a.u.) for no force applied to the maximum force, i.e., 0.35 N (Figure 2g). With increasing load, the intensity profile increased, showing an increased optical transmission. This could be due to the thinning of the sample (under load stress), which makes the sample more transmissive. The diffusion pattern’s length was reduced to 1.5–5 cm range to achieve higher load values per unit length of the replicated diffuser sample. Therefore, for higher values of applied load (reduced diffusion pattern length), measurable fluctuations in intensities around the center at 0° were observed as shown in Figure 2h. This is due to the fact that some light could not scatter and was focused at the center when the dimensions of the structures became too large, causing lower diffusion. Thus, tunable optical diffusion based on the applied load was successfully achieved with a blue laser source of 450 nm. Moreover, the replicated diffuser structure was also tested with other light sources such as green laser (532 nm), red laser (650 nm), and broadband white light (Figure 2i). The intensity distribution profile showed that the diffusion pattern’s length has no relationship with the illuminated incident light wavelength. However, as shown in Figure 2i, measurable intensity distributions, using other light sources such as green laser, red laser, and white light were obtained, indicating the compatibility of the replicated diffuser surface with different wavelengths. However, among all the light sources, the intensity profile of broadband (white light) is more prominent than others; the diffuser structure is compatible with all light sources but a more prominent light response is recorded using broadband. 

The PDMS substrate was grooved with micro/nanostructures, with the dimensions of these structures being comparable to the wavelength of the visible spectrum. These flexible PDMS structures diffused the visible light with a high efficiency and proved to display a wide field of view diffusion. The applied mechanical load altered the average surface morphology of these physical structures and tuned the transmitted light under continuous normal illumination (monochromatic lasers and broadband light source). The tuned diffused light based on external stimuli (mechanical force/stress) was captured from the image screen in front of the sample and correlated with the applied load. The mechanical load applied to the flexible optical diffusers caused the surface structures in the few hundred nanometers to few hundred micrometers range to expand, which in turn changed the transmitted light intensity without interacting with the surface structures and altered the diffusion pattern length across the image screen.

After testing the diffuser, optical experiments were conducted for the flexible PDMS-based periodic gratings. During optical characterizations, a two-dimensional stage along with a weight suspension setup was used as a sample holder to experiment with the polymerized samples for strain sensing. One end of the grating sample was fixed, and the other end was loaded with known weights, such that their suspension stretched the periodic structures. The transmitted diffraction patterns from the grating were captured on an image screen. The pattern contained an intense zero order (non-diffracted central spot) along with diffracted orders. The separation between the non-diffracted zero-order (central spots) and the first-order diffracted spots were taken into account in order to observe the changes in the optical behavior under various mechanical stress conditions.

Optical characterizations of the flexible periodic gratings were performed by transmitting monochromatic light under normal illumination and a far-field experimental setup (Figure 3a). Experimental arrangements were made similar to the experiment for a flexible optical diffuser. Upon illumination with a monochromatic light source, the flexible periodic gratings produced a diffraction pattern on the image screen in transmission mode. The optical response of the periodic gratings was also simulated, with varying grating periodicities and incident wavelengths, as shown in Figure 3b–d. Figure 3b–d shows the squeezing effect of the diffraction pattern in the periodicities range of 0% to 12.5%. When the strain is applied, an increase in the grating periodicity takes place. As a result of such grating extension, the diffraction pattern is squeezed, and the distances between the zero and first-order are reduced.

The experimental investigation for the diffraction distance of replicated periodic gratings was carried out based on the proven simulated response of the squeezing diffraction pattern. The diffraction distance between first-order diffraction and the non-diffracted central spot was measured for red, green, and blue lasers, respectively (Figure 3e). Based on the image screen distance and incident wavelength, the diffraction distances before loading (0 N) were 26, 20.75, and 17.1 cm for red, green, and blue lasers, respectively. However, when the applied load reached the maximum value (0.45 N), all the diffraction distances showed a reducing trend. The red laser with the highest wavelength in response to the maximum load (0.45 N) exhibited the highest reduction in diffraction distance (9.75 cm). A similar reduction trend was observed for the green (7.75 cm) and blue lasers (6.1 cm). In short, all the laser sources showed similar wavelength-dependent reducing trends in the diffraction distance. Furthermore, the highest reduction in diffraction distance indicates the highest decrease in diffraction angle and follows the grating Equation (2). Figure 3f shows a tensile stress–strain diagram of the grating sample upon illumination with three laser sources. The stress–strain curves show that an increased applied load induced more strain in the grating structures, and a similar response was observed for all three light sources. 

**Figure 4:**
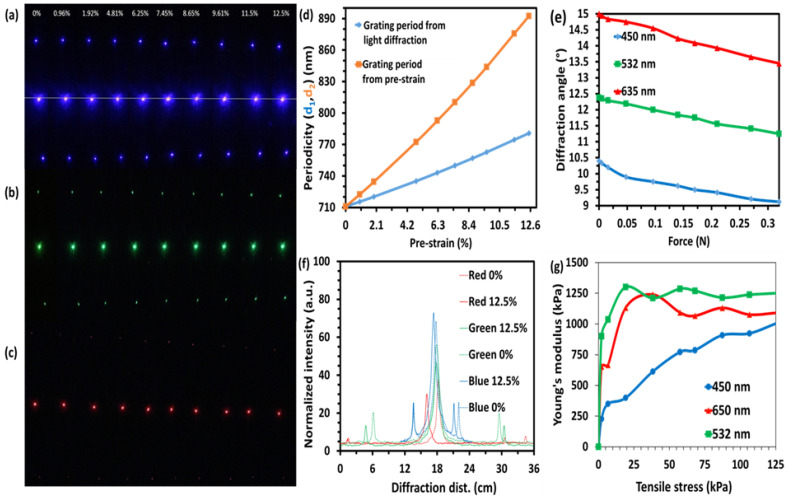
Experimental results of flexible periodic gratings during various light illuminations under various stress/strain levels. (**a**–**c**) Captured diffraction patterns with a digital camera from the image screen obtained by illuminating blue (450 nm), green (532 nm), and red (650 nm) monochromatic lights, respectively, through polymerized periodic gratings, stretched gradually through specific weight suspensions. (**d**) Measured grating period (nm) with respect to applied load (strain) by transmitting a blue (450 nm) light beam through periodic gratings and the grating period calculated from pre-strain. (**e**) Variations in diffraction angles on the diffraction patterns with applied load (force) for blue, green, and red lasers. (**f**) Normalized intensity distribution against diffraction distance (four orders of diffraction) during minimum and maximum stretching (strain) of the flexible periodic gratings under various light illuminations. (**g**) Tensile stress (kPa) versus Young’s modulus (kPa) with blue (450 nm), green (532 nm), and red (650 nm) monochromatic light sources.

The periodic grating sample was strained by applying a variable external load and illuminated by blue, green, and red lasers to investigate the tunable optical response. The diffraction patterns of the grating sample were obtained for the strain levels of 0%, 0.96%, 1.92%, 4.81%, 6.25%, 7.45%, 8.65%, 9.61%, 11.5%, and 12.5% (Figure 4a–c). The increased applied strain led to a shrinkage of the diffraction pattern, indicating reduced diffraction angles. Compared to the red and green lasers, narrower-angle diffraction was observed for the blue laser [63]. After stretching, the first-order angle decreased due to a decrease in diffraction distance (distance between zero and first-order diffraction). The 635 nm red laser caused the least intense first-order diffraction as it entered the grating sample. However, first-order diffraction was seen over the stretching range of 0–12.5% for blue, green, and red lasers (Figure 4a–c).

A linear relationship between the extension ratio of the PDMS block and the periodicity of gratings was found. The periodicities of the gratings (d_2_) calculated by pre-strain have the same trend as the values calculated by experimental data (d_1_) (Figure 4d). Figure 4d shows that the periodicities without any mechanical expansion are around 710.8 nm, as obtained from both methods. However, the growth rate is different for both methods which may be for two main reasons. The first reason is that the grating on the substrate is not filled with the entire substrate. The grating period could not be enlarged with a homogeneous speed during the length of the substrate elongation. Another reason is that the grating could change in the longitudinal direction during the extension of the flexible substrate in the transverse direction. 

The incident monochromatic laser beams gave diffraction patterns on the image screen following Bragg’s law, namely, n·λ = d·sinθ, where n is an integer signifying the diffraction order, d is the groove spacing or period of the grating, and θ depicts the diffraction angle. The first-order diffraction angle can be deduced from the experimental observation by θ = sin^−1^(l_1_/(l_1_^2^+l_2_^2^)^1/2^), where l_1_ and l_2_ represent the first-order interspace and distance between the grating and image screen, respectively [58,59,61,64]. Consequently, the change in the grating period, and therefore in length against any load, can be deduced by taking the difference between the upstretched condition, namely, d = λ·l_1_/(l_1_^2^+l_2_^2^)^1/2^, and the stretched condition, for which di = λ·l_i_/(l_i_^2^+l_22_)^1/2^, where the subscript i denotes the ith load suspended to the flexible grating [65]. 

The diffraction angle is the separation between diffracted and non-diffracted spots found directly proportional to the applied load (N) as predicted by simulations. The diffraction angles and the periodicities of the gratings were found to be inversely proportional to each other. For each increment in the value of applied force to expand the grating’s period, the diffraction angle decreased from the wavelength of the illuminated laser, i.e., all wavelengths followed the same trend (Figure 4e). Thus, the red light diffracted at the highest angle of 15° for the no-load (0N) application and 13.45° for the maximum load (0.35 N). In contrast, blue being the lowest wavelength in the visible spectrum diffracted at the lowest angles of 10.4° for no load (0N) and 9.12° for the maximum load (0.35 N), as shown in Figure 4e. The diffraction angle consistently depended on the wavelength of the illuminated light, as well as on the periodicities of the grating structure. The change in diffraction angle could also be expressed as a function of applied stress in the form of the degree of expansion of flexible periodic gratings. The red laser (650 nm) has the largest wavelength within the visible spectrum, hence it was diffracted at the largest angles throughout, whereas blue (450 nm) has the lowest wavelength within the spectrum and diffracted at the lowest angles under all stress levels. However, a similar trend for all three wavelengths is yielded. The change in diffraction angles for lower applied force (less than 0.1 N) showed a slightly less steep response, whereas, above 0.1 N, it showed a consistent decline up to its elastic limit because of the threshold force which is the minimum force required to start expansion.

The periodic grating structure also enabled the investigation of mechanically adjustable optical intensity information (Figure 4f). Pliable PDMS grating periodicities were increased based on the four orders of diffraction including non-diffraction order in size upon application of load (strain) from minimum (0%) and maximum (12.5%) that caused variations in transmitted light intensity distribution. The increased periodicity means that the distance between the two slits increased, and the transmitted light would have to pass through a stretched polymer material with a different thickness than the parent one, causing retardation in the intensity of transmitted light for all the diffraction orders depending on the incident wavelengths [66]. However, a slight increase in the intensity of the non-diffracted spot (zero-order diffraction) for the blue (450 nm) laser can be seen and might be attributed to higher transmission through a thinner polymer sample.

Young’s modulus was also measured from the stress–strain curve of the grating structure. Using the area of the cross-section and net applied force, the tensile stress was determined (Figure 4g). The average of measured values was taken to calculate the cross-sectional area to get an accurate result. The highest Young’s modulus was created by the chromatic green laser (0–1250 kPa) in the strain range of (0–12.5%). In contrast, the monochromatic blue laser created the lowest Young’s modulus in the range of 0–1004.96 kPa. Figure 4g shows that green and red lasers followed a similar trend for tensile stress against Young’s modulus. A sudden rise in Young’s modulus values at the beginning and peak values of 1210.53 kPa and 1239.45 kPa by green and red lasers, respectively, around 40 kPa tensile stress are observed. After achieving the peak value, the graphs have a smaller decline and maintain a relatively stable slow rise. In contrast, Young’s modulus for the blue (450 nm) wavelength shows rapid growth at the beginning and achieves a relatively slower growth until the tensile stress reaches 20 kPa. However, the blue laser peak value of 613.33 kPa was also observed around 40 kPa tensile stress. It could be inferred that the main parameter affecting Young’s modulus is applied strain (change in optical response, i.e., diffraction angle). Young’s modulus of PDMS is in the range of 360–870 kPa, and experimental modulus results demonstrate that all the values are affected by the applied strain and are in a reasonable range.

## 4. Conclusions and Perspective

We have successfully demonstrated the mechanically tunable optical properties of the polymerized optical devices including periodic gratings and optical diffusers based on external stimuli (applied load). Compared to rigid conventional nano/microstructures, tunable optical diffusion and diffraction angles were achieved using flexible optical diffusing surfaces and periodic gratings, respectively. Moreover, the uses of rigid optical devices are limited due to their constant dimensions, whereas flexible optical devices can change their structures (dimensions) in response to external stimuli by yielding tunable optical responses. As such devices are composed of passive components eliminating the use of electronics or power supply, the PDMS-based optical strain sensors described in the present work are simple, low cost, easy to fabricate, and feasible to operate in complex environments. The sensitivity of polymerized sensors could be customized by adding impurities into the PDMS solution during fabrication or treating them with polishes, paints, and coatings with selective metallic/nonmetallic components to obtain desirable optical/mechanical properties. Our findings are as follows:

An inverse relationship between applied force and diffraction angle of transmitted light was demonstrated during optical characterizations of flexible periodic gratings to be used as a strain sensor.

In contrast, a direct relationship was observed between the diffusion field and applied load during the optical characterization of polymerized optical diffusers under various stress levels.

Recorded tunable optical responses were correlated with tensile stress and were in good agreement with elastic modulus values. Sensors based on soft, flexible nano/microstructures are reliable and may have applications in remote-sensing as strain sensors.

It is anticipated that instead of mechanical expansion/compression, similar results could be achieved under thermal expansion/compression.

The performance of PDMS-based sensors could be affected by environmental conditions such as humidity, temperature, and swelling characteristics.

## Data Availability

The data presented in this study are available on request from the corresponding authors.
